# Efficacy and Safety of Percutaneous Pedicle Screw Fixation for Neurologically Intact Thoracolumbar Burst Fractures: A Prospective Interventional Study

**DOI:** 10.7759/cureus.113263

**Published:** 2026-07-23

**Authors:** Deepak K Saini, Krishna Kumar, Gnanaprakash Gurusamy, Ijack Debbarma, Ashish Jaiman, Tankeshwar Boruah

**Affiliations:** 1 Orthopedics, Maharishi Chyawan Government Medical College and Rao Tula Ram Hospital, Narnaul, IND; 2 Orthopedics, Vardhman Mahavir Medical College and Safdarjung Hospital, New Delhi, IND; 3 Spine Surgery, Ganga Medical Centre and Hospitals Private Limited, Coimbatore, IND; 4 Orthopedics, Agartala Government Medical College (AGMC) and Govind Ballabh Pant (GBP) Hospital, Agartala, IND

**Keywords:** burst fracture, fluoroscopy, gertzbein-robbins, minimally invasive spine surgery, percutaneous pedicle screw fixation, thoracolumbar fracture

## Abstract

Background: Open posterior short-segment pedicle instrumentation is an established treatment for unstable thoracolumbar fractures but carries approach-related morbidity from paraspinal muscle stripping. Percutaneous pedicle screw fixation (PPSF) aims to provide comparable stabilization with reduced soft-tissue trauma. We evaluated the perioperative profile, radiographic correction, and screw-placement accuracy of PPSF performed under single conventional fluoroscopy in neurologically intact patients.

Methods: In this prospective, single-arm interventional study, 46 consecutive patients aged 18-50 years with AO Spine type A3/A4 thoracolumbar fractures and intact neurology (American Spinal Injury Association (ASIA) grade E) underwent short-segment PPSF over an 18-month period at a tertiary public hospital. Operative time, intraoperative blood loss, visual analog scale (VAS) pain score, length of hospital stay, sagittal Cobb angle, and anterior vertebral body height (AVBH) were recorded. Screw placement was graded on postoperative CT using the Gertzbein-Robbins classification. Preoperative and postoperative radiographic parameters were compared using paired analysis.

Results: Mean age was 30.8±8.8 years; 28 patients (60.9%) were female. The most common mechanism of injury was a fall from height (65.2%), and the most commonly affected level was L2 (54.3%). Mean operative time was 108.2±12.1 minutes, and mean blood loss was 102.6±16.1 mL. Mean length of hospital stay was 2.6±0.7 days, and mean VAS score decreased from 4.4±0.6 (day 0) to 2.6±0.7 (day 3). Cobb angle improved from 18.9±5.9° to 8.7±5.9°, and AVBH improved from 55.3±11.0% to 74.3±10.4% (both p<0.01). Of 184 screws, 173 (94.0%) were Gertzbein-Robbins grade A, whereas 11 (6.0%) were malpositioned (grades B-D), with none causing neurovascular injury. There were no infections, implant failures, or screw revisions.

Conclusion: PPSF is a safe and minimally invasive technique that demonstrated favorable immediate perioperative and radiographic outcomes, with high pedicle screw placement accuracy and no early procedure-related complications in selected neurologically intact patients with thoracolumbar fractures. Further prospective comparative studies with longer follow-up are required to establish its long-term clinical outcomes and comparative effectiveness.

## Introduction

Thoracolumbar fractures are among the most common spinal injuries, with burst fractures predominating at the thoracolumbar junction. The management of unstable burst and flexion-distraction injuries in patients without neurological deficit remains debated, with both operative and non-operative strategies in use. Non-operative management with bracing or recumbency can yield acceptable outcomes but has been associated with residual kyphosis, prolonged immobilization, and late neurological deterioration in a subset of patients [1].

The goals of treatment are to restore and maintain sagittal alignment and vertebral height, achieve fracture healing, and, where possible, preserve adjacent-segment motion [2]. Surgical stabilization provides more reliable restoration of alignment, vertebral height, and canal dimensions than conservative care, and posterior short-segment pedicle instrumentation is the most widely used construct worldwide [3-6].

However, the conventional open posterior approach requires extensive paraspinal exposure and is associated with significant blood loss, infection risk, and iatrogenic muscle denervation [5,6].

Percutaneous pedicle screw fixation (PPSF), introduced by Magerl and later refined for the lumbar spine, was developed to provide equivalent posterior stabilization while limiting soft-tissue trauma [7,8]. Compared with open fixation, PPSF has been associated with less paraspinal muscle damage, reduced blood loss, less postoperative pain, and a shorter hospital stay [9,10]. Its principal trade-offs include dependence on intraoperative imaging, with attendant radiation exposure, the absence of formal arthrodesis and decompression, and a recognized learning curve.

Most reported PPSF series originate from centers using biplanar imaging or navigation. Evidence from public tertiary centers using a single conventional C-arm is more limited. This study evaluated the perioperative profile, immediate radiographic correction, and pedicle screw placement accuracy of PPSF performed under single conventional fluoroscopy in neurologically intact patients with thoracolumbar fractures.

Aim and objectives

The aim of this study was to evaluate the efficacy and safety of PPSF for thoracolumbar fractures. The specific objectives were to assess (i) postoperative hospital stay, postoperative pain, kyphotic (Cobb) angle correction, and vertebral body height restoration; and (ii) pedicle screw placement accuracy, operative time, and intraoperative blood loss.

## Materials and methods

Study design and setting

This was a prospective, single-arm, hospital-based interventional study conducted over an 18-month period from October 2019 to March 2021 at a tertiary care center. The study was approved by the Institutional Ethics Committee, and written informed consent was obtained from all patients or their legally authorized representatives. There was no concurrent open-surgery comparison group.

Sample size

Taking the proportion of satisfactory outcomes after PPSF as 86.1% from Ni et al. [11], a sample size of 46 was calculated for a margin of error of 10% at a 5% level of significance using the formula n≥p(1−p)/(ME/Zα)².

Participants

Patients aged 18-50 years of either sex with AO Spine type A3 or A4 thoracolumbar fractures or type B1/B2 flexion-distraction (Chance) injuries and intact neurology (American Spinal Injury Association (ASIA) Impairment Scale grade E) were included in the study. Patients with any neurological deficit, fracture types other than those specified, spinal tumors or suspected pathological fractures, severe osteoporosis, or spinal tuberculosis were excluded.

Preoperative assessment

Plain anteroposterior and lateral radiographs were used to measure the sagittal Cobb angle and anterior vertebral body height (AVBH). The sagittal Cobb angle was measured between the superior endplate of the vertebra above and the inferior endplate of the vertebra below the fractured vertebra, while AVBH was calculated as the percentage of the anterior height of the fractured vertebral body relative to the estimated normal height derived from the adjacent vertebrae. Computed tomography (CT) was used to assess fracture comminution and fragment apposition, whereas magnetic resonance imaging (MRI) was used to evaluate the posterior ligamentous complex and spinal cord. Neurological status was documented using the ASIA Impairment Scale [12-14].

Surgical technique

**Figure 1:**
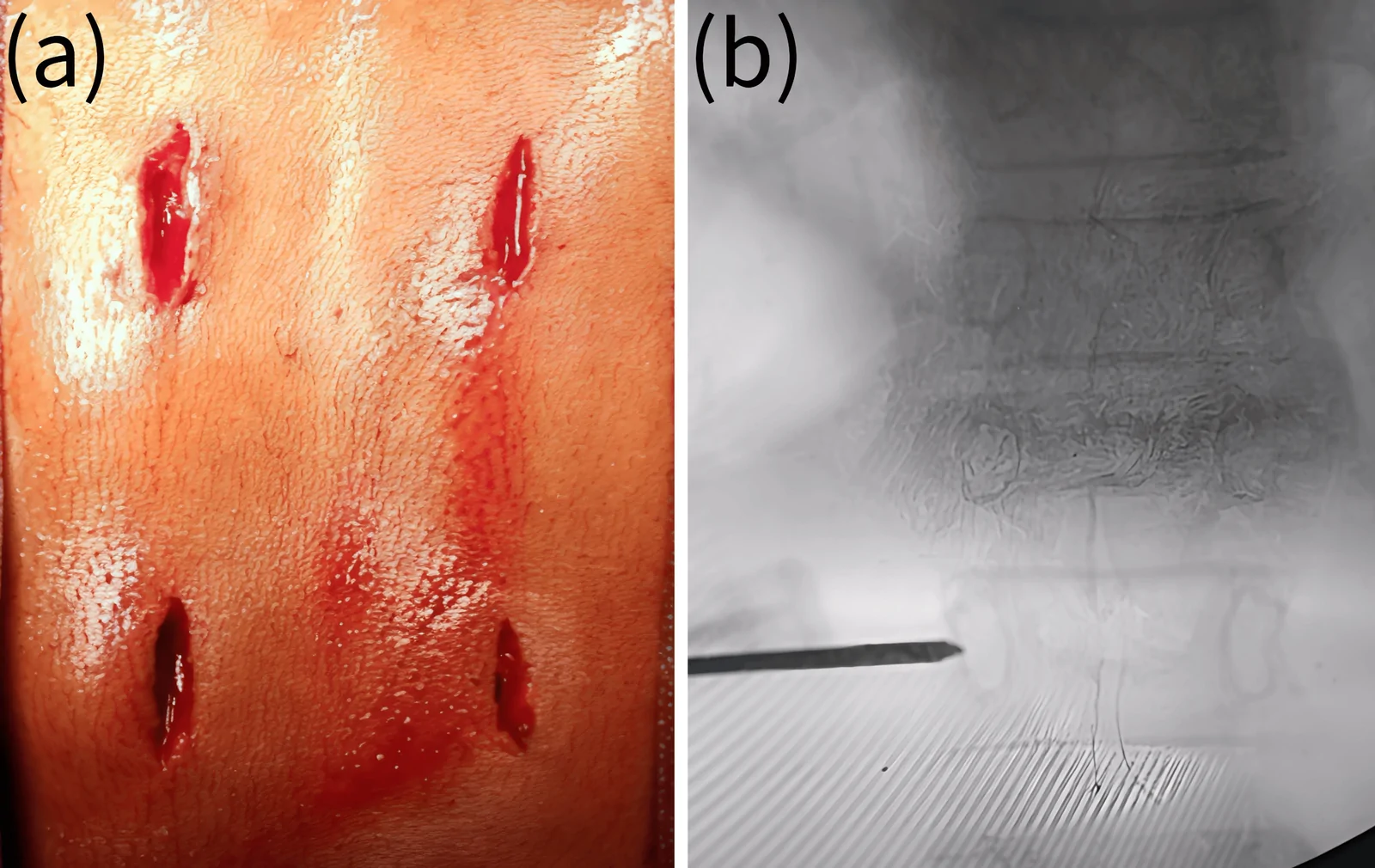
(a) Skin incision. (b) Entry point of the Jamshidi needle

After induction of general anesthesia, patients were carefully positioned prone on a radiolucent operating table with chest and pelvic bolsters to promote lordosis, with appropriate padding and meticulous protection of the abdomen, external genitalia, pressure points, and endotracheal tube. Short-segment percutaneous fixation was performed using pedicle screws inserted one level above and one level below the fractured vertebra, connected with contoured rods under fluoroscopic guidance using a conventional C-arm. A single conventional C-arm was used to obtain true anteroposterior and lateral projections aligned with the vertebral endplates. Skin entry points were marked 1-2 cm lateral to the target pedicle (2-3 o'clock on the right and 9-10 o'clock on the left) to permit a convergent trajectory (Figure 1).

**Figure 2:**
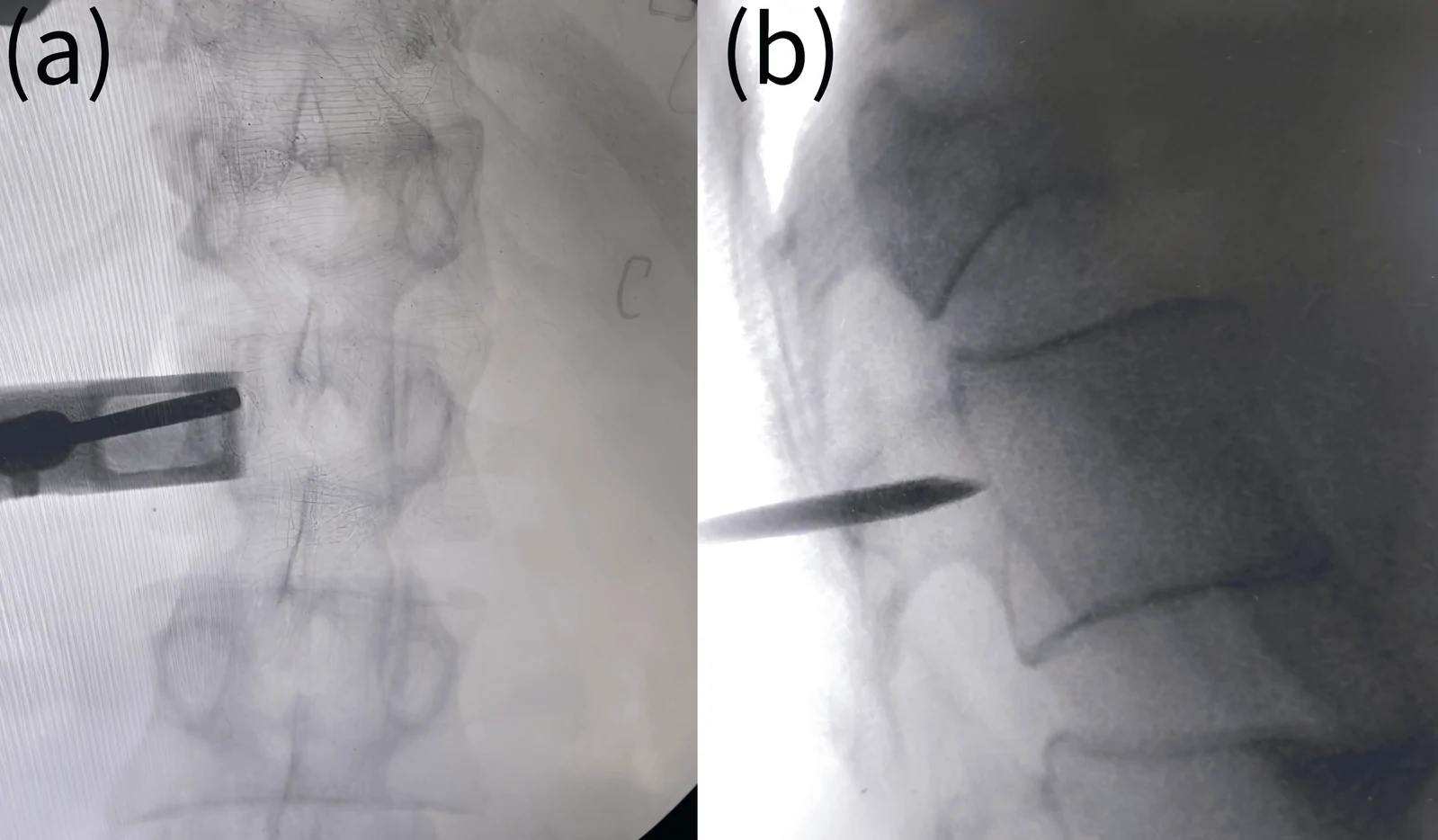
(a) Tip of the Jamshidi needle at the center of the pedicle. (b) Tip of the Jamshidi needle approaching the posterior wall of the vertebral body

A Jamshidi needle was advanced to the base of the pedicle under intermittent fluoroscopic guidance. A guidewire was then passed into the vertebral body, taking care not to breach the anterior cortex (Figure 2).

**Figure 3:**
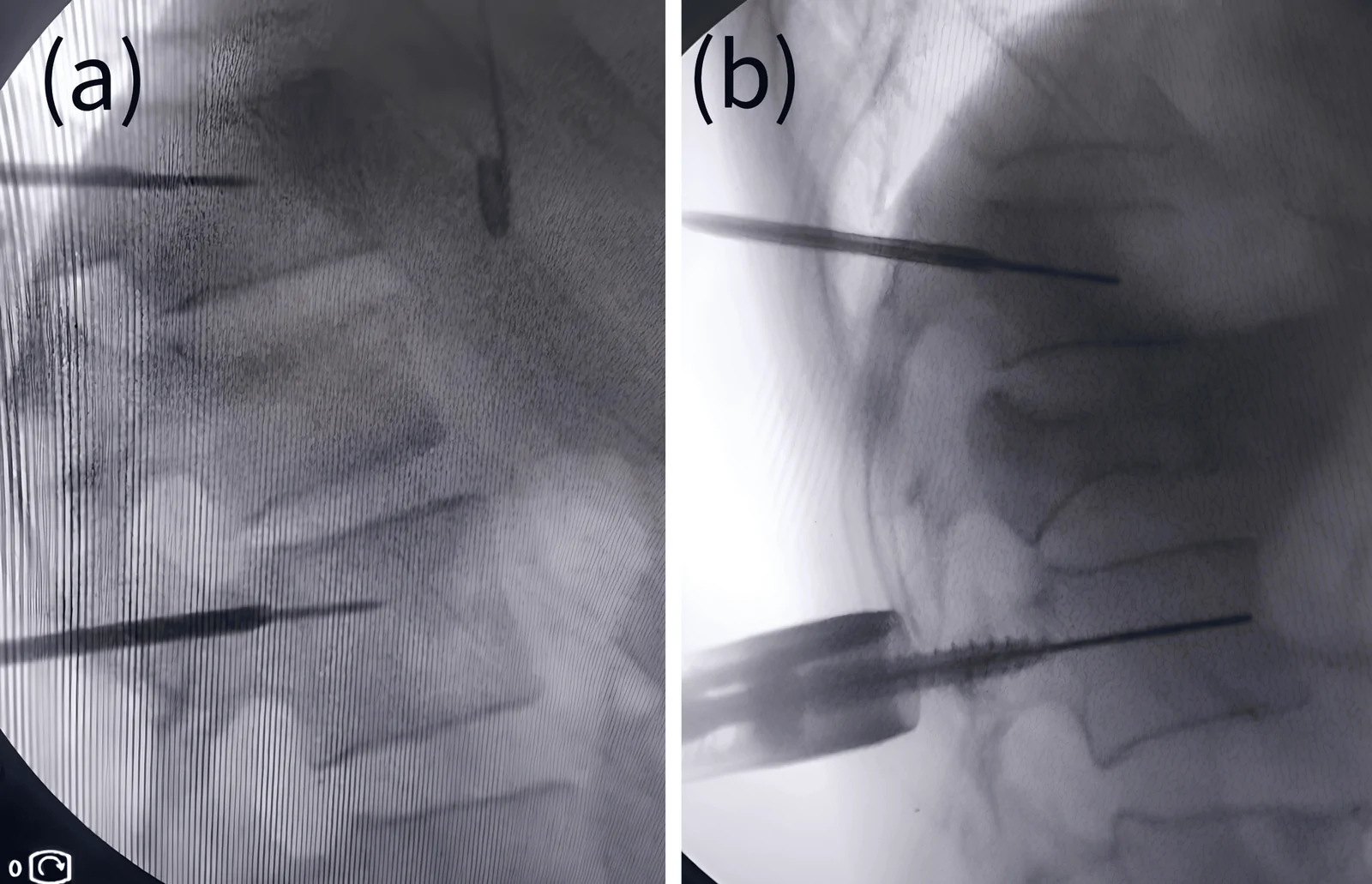
(a) Guidewire inserted. (b) Pedicle screw insertion over the guidewire

**Figure 4:**
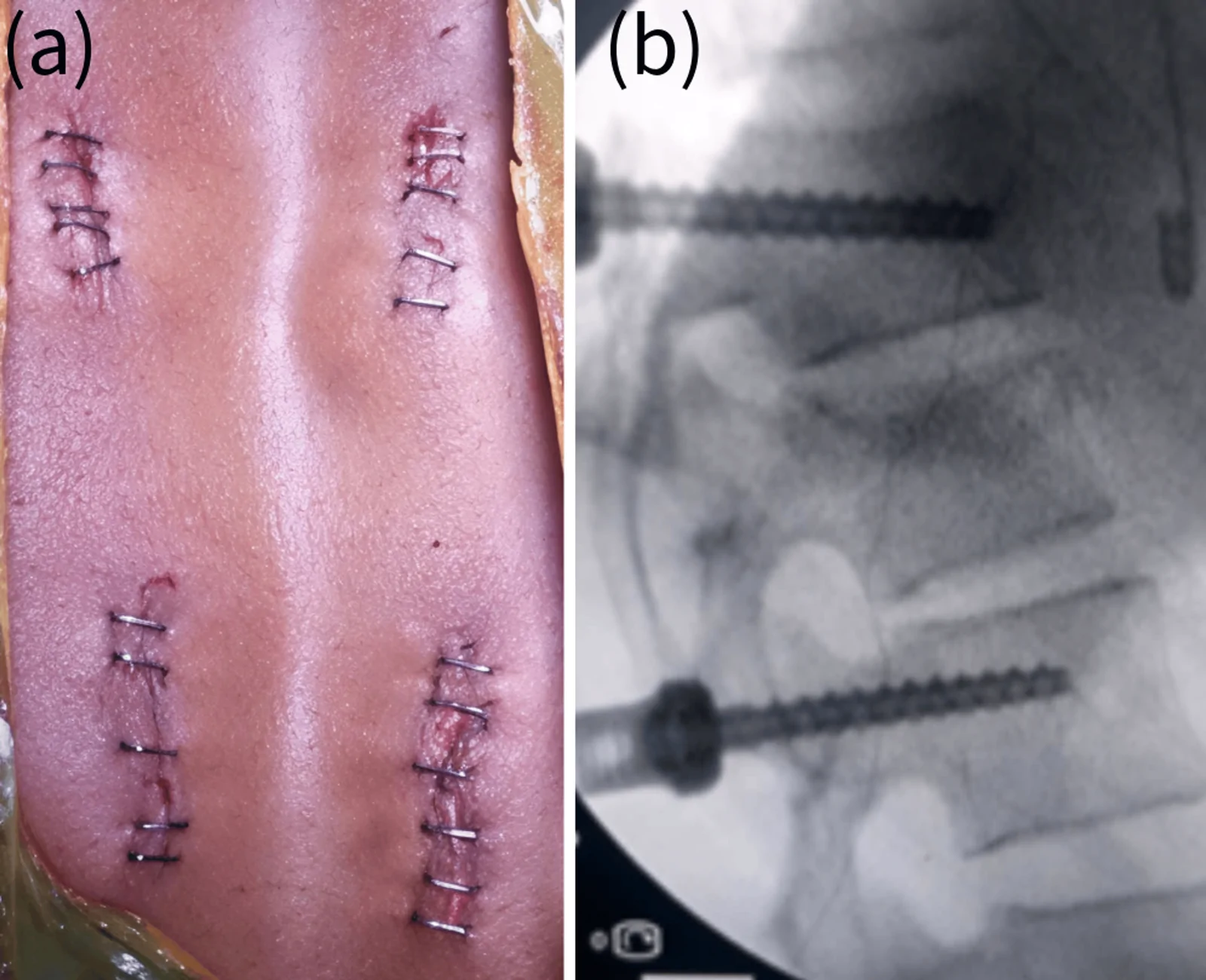
(a) Skin closure. (b) Final position of the pedicle screws

After sequential dilation and cannulated tapping, cannulated pedicle screws were inserted over the guidewires, followed by placement of the contoured rods (Figure 3). The wounds were closed in layers (Figure 4).

Postoperative assessment and outcomes

Postoperative radiographs were obtained to remeasure the Cobb angle and AVBH, and CT was performed to grade screw placement using the Gertzbein-Robbins classification with 2-mm pedicle breach increments (grade A, fully contained; grade B, 0-2 mm; grade C, 2-4 mm; grade D, 4-6 mm) [15]. Pain was assessed using a 0-10 visual analog scale (VAS) on the day of surgery and on postoperative day 3. Operative time, intraoperative blood loss, length of hospital stay, and complications (neurological injury, infection, implant failure, and revision surgery) were recorded. All patients received the institutional postoperative protocol, including early mobilization as tolerated, appropriate analgesia, brace support when indicated, and physiotherapy before discharge.

Statistical analysis

Statistical analysis was performed using SPSS Statistics version 21.0 (IBM Corp., Armonk, NY, USA). Continuous variables were expressed as mean±standard deviation (SD) and range, while categorical variables were presented as frequencies and percentages. Normality of continuous variables was assessed using the Shapiro-Wilk test. Normally distributed paired variables were compared using the paired Student's t-test, whereas non-normally distributed paired variables were compared using the Wilcoxon signed-rank test. Categorical variables were summarized descriptively without inferential testing because the study lacked a comparison group. A p-value <0.05 was considered statistically significant.

## Results

Baseline demographic and injury characteristics 

Forty-six patients met the inclusion criteria. The mean age was 30.8±8.8 years (range, 18-50 years); 28 (60.9%) were female, giving a female-to-male ratio of 1.55:1. The most common mechanism of injury was a fall from height (30; 65.2%), followed by falls from stairs (9; 19.6%) and road traffic accidents (7; 15.2%), representing high-energy trauma. The most frequently involved level was L2 (25; 54.3%), and all fractures were AO type A3 (20; 43.5%) or A4 (26; 56.5%); no type B injuries occurred during the study period. Calcaneal fractures coexisted in 13 patients (28.3%), of whom nine had bilateral fractures. Baseline characteristics are summarized in Table 1.

**Table 1:** Baseline demographic and injury characteristics (n=46) ^#^Fracture Categorical variables were presented as frequencies and percentages and summarized descriptively without inferential testing because the study lacked a comparison group.

Characteristics	Category	n (%)
Age group (years)	≤20	5 (10.9)
	21-30	19 (41.3)
	31-40	15 (32.6)
	41-50	7 (15.2)
Sex	Female	28 (60.9)
	Male	18 (39.1)
Mechanism	Fall from height	30 (65.2)
	Fall from stairs	9 (19.6)
	Road traffic accident	7 (15.2)
Fracture level	D12	4 (8.7)
	L1	13 (28.3)
	L2	25 (54.3)
	L3	4 (8.7)
AO type	A3	20 (43.5)
	A4	26 (56.5)
Associated injury	Bilateral calcaneal^#^	9 (19.6)
	Unilateral calcaneal^#^	4 (8.7)
	None	33 (71.7)

Perioperative outcomes

Mean operative time was 108.2±12.1 minutes (range, 90-150), and mean intraoperative blood loss was 102.6±16.1 mL (range, 70-150). Mean length of hospital stay was 2.6±0.7 days (range, 2-4). Mean VAS pain score decreased from 4.4±0.6 on the day of surgery to 2.6±0.7 on postoperative day 3 (Table 2).

**Table 2:** Perioperative parameters (n=46) Continuous variables were expressed as mean±SD and range. VAS: visual analog scale; SD: standard deviation

Parameter	Mean±SD	Range
Operative time (min)	108.2±12.1	90-150
Intraoperative blood loss (mL)	102.6±16.1	70-150
VAS, day of surgery	4.4±0.6	4-6
VAS, postoperative day 3	2.6±0.7	2-4
Length of stay (days)	2.6±0.7	2-4

Radiographic correction

The sagittal Cobb angle improved from a preoperative mean of 18.9±5.9° to a postoperative mean of 8.7±5.9°, representing a mean correction of 10.2±4.2°. AVBH increased from 55.3±11.0% to 74.3±10.4% of normal, representing a mean restoration of 18.9±7.5%. Both changes were statistically significant (p<0.01) (Table 3). These measurements reflect the immediate postoperative period.

**Table 3:** Radiographic parameters before and after surgery Normally distributed paired variables were compared using the paired Student's t-test. A p-value <0.05 was considered statistically significant. SD: standard deviation; VBH: vertebral body height; df: degrees of freedom

Parameter	Preoperative (mean±SD)	Postoperative (mean±SD)	Mean difference±SD	Paired t-value	df	P-value
Cobb angle (°)	18.9±5.9	8.7±5.9	10.2±4.2	16.47	45	<0.01
Anterior VBH (% normal)	55.3±11.0	74.3±10.4	18.9±7.5	17.10	45	<0.01

Screw placement accuracy

A total of 184 screws were placed (92 on each side). On postoperative CT, 173 screws (94.0%) were classified as Gertzbein-Robbins grade A (fully contained). Eleven screws (6.0%) were malpositioned: nine (4.9%) were grade B, one (0.5%) was grade C, and one (0.5%) was grade D, distributed between D12 and L3. No malpositioned screw caused neurovascular injury, and no screw required revision (Table 4).

**Table 4:** Gertzbein-Robbins grade of pedicle screw placement (n=184 screws)

Grade (breach)	Screws, n	%
A (contained)	173	94.0
B (0-2 mm)	9	4.9
C (2-4 mm)	1	0.5
D (4-6 mm)	1	0.5
Total	184	100.0

Complications

No patient developed a new neurological deficit, surgical-site infection, or implant failure during the study period, and no screw revision was performed.

## Discussion

In this single-center series of 46 neurologically intact patients with thoracolumbar burst fractures, PPSF performed using a single conventional C-arm achieved high pedicle screw placement accuracy (94.0% grade A), low blood loss (mean, 103 mL), a short hospital stay (mean, 2.6 days), and significant immediate correction of both kyphosis and AVBH, with no recorded complications. These findings are broadly consistent with the published PPSF literature.

The mean operative time of 108 minutes was longer than that reported in several PPSF series, for example, 78 minutes in Ni et al. and 81 minutes in Silva et al., most plausibly because a single conventional fluoroscope was used rather than biplanar imaging or navigation [11,16]. Blood loss (103 mL) and length of hospital stay (2.6 days) were nonetheless favorable and within or below the range reported for open short-segment fixation, in which blood loss and hospital stay are typically substantially greater. Because the present study had no internal control group, these comparisons are descriptive only; no statistical comparison between PPSF and open surgery was performed.

Kyphosis correction (10.2°) and AVBH restoration (18.9%) were significant and comparable to those reported by Silva et al., Ni et al., and Yang et al. [11,16,17]. Postoperative pain scores decreased rapidly, consistent with the reduced paraspinal dissection inherent to the percutaneous approach. A screw malposition rate of 6.0% closely accords with reported rates of 5.9%-6.7% using fluoroscopy-guided percutaneous insertion [11,17,18]. None of the malpositioned screws in this series produced clinical sequelae, supporting the safety of the technique even without tactile pedicle feedback.

An unexpected finding was the female predominance (60.9%), which contrasts with most thoracolumbar trauma series, in which male patients predominate [19]. The most likely explanation is selection bias: by excluding patients with neurological deficits, who are more often male and more often injured in high-energy mechanisms such as road traffic accidents, the cohort was enriched for lower-energy mechanisms, with falls from height predominating. This interpretation is plausible but cannot be confirmed from the present data and should be regarded as hypothesis-generating.

The principal trade-offs of PPSF are the absence of formal arthrodesis and canal decompression and increased radiation exposure. The stability of a burst fracture managed without fusion depends on bony healing and remodeling; for more unstable injuries (e.g., type B2/C) or those with neurological compromise, open decompression and fusion remain indicated. The reported screw malposition rate for the open technique using anatomical landmarks is 10%-20% [20,21]. The lower rate observed in the present study is reassuring for image-guided percutaneous placement.

Although the immediate outcomes observed in this study are encouraging, the absence of a comparison group and long-term follow-up limit conclusions regarding the durability of these results and the comparative effectiveness of PPSF.

Limitations

This study has several limitations. It was a single-arm series with no open-surgery comparison group, so relative effectiveness cannot be inferred. The sample size was modest. Critically, radiographic and clinical outcomes were assessed only in the immediate postoperative period; no defined follow-up interval was reported, so maintenance of correction, loss of reduction, fracture union, implant behavior, and functional recovery over time were not evaluated. Outcomes were also limited to a single fracture pattern (A3/A4 fractures with intact neurology), limiting generalizability. The statistical test used for paired comparisons was not specified in the source data and is assumed to be a paired t-test. These constraints should temper interpretation of the efficacy findings, which are best viewed as evidence of safe, accurate fixation with good immediate correction rather than proof of durable clinical benefit. Additional limitations include the lack of blinded radiographic assessment, which may have introduced observer bias, and the absence of validated functional outcome measures, such as the Oswestry Disability Index or Short Form-36 Health Survey (SF-36), precluding a comprehensive evaluation of postoperative functional recovery and health-related quality of life.

## Conclusions

PPSF is a safe, effective, and minimally invasive treatment option for neurologically intact patients with thoracolumbar vertebral fractures. In this prospective study of 46 patients, the technique was associated with low intraoperative blood loss, acceptable operative time, reduced postoperative pain, a short hospital stay, significant correction of kyphotic deformity, and restoration of AVBH. Furthermore, postoperative CT evaluation demonstrated high pedicle screw placement accuracy, with no neurovascular complications related to screw malposition, confirming the safety of the procedure.

The present study supports the immediate technical safety and favorable perioperative outcomes of PPSF in selected AO type A3 and A4 thoracolumbar fractures without neurological deficit. By minimizing soft-tissue injury while providing reliable spinal stabilization and satisfactory radiologic outcomes, this technique facilitates early postoperative recovery and may reduce approach-related morbidity. However, no conclusions can be drawn regarding the long-term maintenance of deformity correction, implant longevity, functional recovery, or late complications. The absence of a comparison group precludes assessment of comparative effectiveness. Nevertheless, larger prospective comparative studies with longer follow-up are required to evaluate the long-term clinical efficacy, durability of deformity correction, implant-related complications, the need for implant removal, and the comparative effectiveness of PPSF.
